# Twin Small RNAs and Divergent Fates: The Expansive Regulatory Networks of OmrA and OmrB

**DOI:** 10.3390/ijms262311713

**Published:** 2025-12-03

**Authors:** Julia Konarska, Karolina Jaworska, Paulina Lipska, Adrianna Raczkowska

**Affiliations:** University of Warsaw, Faculty of Biology, Institute of Microbiology, Department of Molecular Microbiology, Miecznikowa 1, 02-096 Warsaw, Poland; j.konarska2@uw.edu.pl (J.K.); k.jaworska3@uw.edu.pl (K.J.); p.lipska3@student.uw.edu.pl (P.L.)

**Keywords:** sRNA, OmrA, OmrB, chaperone Hfq, virulence, iron homeostasis, biofilm, motility

## Abstract

Small RNAs (sRNAs) have emerged as key regulators of bacterial physiology, enabling rapid adaptation to environmental changes through post-transcriptional control. The homologous sRNAs OmrA and OmrB, conserved in *Escherichia coli* and other *Enterobacterales*, share sequence similarity yet differ in expression dynamics and functional outputs. Both act by base-pairing with target mRNAs, a process facilitated by the RNA chaperone Hfq, which promotes duplex formation and influences RNA stability. In some contexts, regulation also depends on RelA, which stabilizes Hfq-RNA complexes and links OmrA/B activity to the stringent response. Together, OmrA and OmrB modulate outer membrane protein synthesis, motility, biofilm formation, iron uptake, and virulence. OmrA is preferentially induced under nutrient limitation and integrates into the σ^S^ stress regulon, whereas OmrB is more broadly expressed and can engage in context-dependent mechanisms such as target sequestration. This review highlights the molecular mechanisms of OmrA/B regulation and their contribution to global sRNA-mediated regulatory networks that coordinate bacterial adaptation and pathogenicity.

## 1. Introduction

Small noncoding RNAs (sRNAs), usually between ~50 and 200 nucleotides long, have become key players in bacterial post-transcriptional regulation. Bacterial sRNAs are generally classified into two groups: *cis*-encoded antisense RNAs, which are transcribed from the DNA strand opposite their target gene and show strong complementarity, and *trans*-encoded sRNAs, which are encoded on separate genomic loci and bind imperfectly to multiple mRNAs through short seed regions [1,2]. *Trans*-acting sRNAs commonly regulate gene expression by blocking or exposing ribosome-binding sites to repress or activate translation, by promoting or preventing RNase E-mediated decay, or by influencing transcription termination and mRNA processing [3,4,5]. Functionally, sRNAs are involved in a variety of processes, such as maintaining envelope integrity and regulating metabolic homeostasis, quorum sensing, virulence and host interaction, stress adaptation, motility and biofilm formation [6,7,8,9,10,11].

Computational analyses have identified hundreds of these noncoding regulatory RNAs in *Escherichia coli*, but only around 80 have been confirmed through experimental validation [12]. OmrA and OmrB are two *trans*-acting homologous small RNAs (sRNAs) originally discovered in non-pathogenic *E. coli*, encoded within an approximately 600-base pair intergenic region located between the *aas* and *galR* genes. These two sRNAs are the product of an ancestral gene duplication event and share a high degree of sequence similarity. OmrA is 88 nucleotides long, while OmrB is slightly shorter at 82 nucleotides. They are nearly identical at their 5′ and 3′ terminal regions, with divergence occurring after the 21st nucleotide; the middle sections of OmrA/B are different—32 nucleotides long in OmrA and 26 in OmrB. The conserved terminal sequences are essential for target recognition and binding, and their conservation across other bacterial species suggests that these regions fulfill important and evolutionarily preserved functions [13,14,15]. This arrangement is preserved in most, though not all, *Enterobacterales*. While this structural similarity implies functional overlap, differences in expression dynamics have been observed. OmrA expression peaks during the late stationary phase, whereas OmrB shows more variable expression patterns [16]. Additionally, environmental factors such as temperature or osmolarity further influence their differential expression [17]. OmrA/B typically use their 5′ seed region to pair near the Shine–Dalgarno/start codon of target mRNAs, therefore preventing ribosome binding, a classical mechanism for translational repression [18]. For many bacterial *trans*-encoded sRNAs, including OmrA/B, effective function depends on their association with RNA chaperone Hfq, which not only protects them from ribonuclease-mediated degradation but also promotes their interaction with target mRNAs. By binding to both the sRNA and the target transcript, Hfq supports imperfect base-pairing that would otherwise be inefficient or unstable. In this way, Hfq ensures that sRNAs effectively repress or activate gene expression after transcription [19,20,21].

Comparative genomic studies have confirmed the presence of OmrA/B across many *Enterobacterales*, although the conservation of OmrB is more variable. Skippington and Ragan (2012) [22] investigate the evolutionary origin, regulatory functions, conservation, and variability of OmrA/B in *E. coli* and *Shigella*. Both sRNAs have been shown to regulate nearly identical targets, a redundancy that may give an evolutionary advantage by enabling tighter control of regulator abundance and mRNA targets. OmrA is conserved across all 27 *E. coli* and *Shigella* genomes analyzed, and the intergenic region encoding *omrA* and *omrB* is preserved in many *Enterobacterales* genomes. By contrast, OmrB is absent from both strains of *S. boydii* examined, demonstrating that this redundancy is dispensable in certain lineages. Phylogenetically, OmrB is classified as a variable sRNA and inferred to be paraphyletic, consistent with gene loss during *E. coli-Shigella* evolution. Given its substantial overlap in regulatory targets with OmrA, OmrB represents an unusual case of a variable sRNA modulating core genes. Whereas most variable sRNAs interact with only one or two targets, OmrA/B is exceptional in acting on multiple targets, a feature more typical of broadly conserved sRNAs [2,22].

## 2. Regulation of *omrA*/*B* Expression

### 2.1. EnvZ/OmpR Two-Component System

Expression of OmrA/B is induced by environmental signals through the EnvZ/OmpR two-component system, which directly activates their transcription. Two-component systems are widespread in bacteria and mediate adaptation to environmental changes by a sensor kinase and a response regulator. When the sensor detects a stimulus, it undergoes autophosphorylation and then transfers the phosphate group to the regulator protein, which in turn controls the expression of target genes. EnvZ, an inner membrane-associated sensor kinase, phosphorylates or dephosphorylates the response regulator OmpR, thereby modulating its activity. OmpR is a global transcriptional regulator that controls a wide range of genes involved in outer membrane permeability (e.g., *ompF* and *ompC*, encoding major porins), stress adaptation and biofilm formation (e.g., *csgD*, the master regulator of curli and extracellular matrix production), motility (e.g., *flhDC*, the flagellar master regulator), and metabolic/transport processes (e.g., *narU*, a nitrate/nitrite transporter, and *tppB*, a tripeptide permease), thereby coordinating bacterial responses to osmotic, environmental, and host-associated signals. Genome-wide analyses in *E. coli* have identified at least 37 genes within 24 transcriptional units as part of the OmpR regulon under osmotic stress conditions [23,24].

EnvZ becomes activated under high-osmolarity conditions. At low osmolarity, when phosphorylated OmpR (OmpR-P) levels are low, the *ompF* gene is transcribed, producing a porin with a relatively large pore size. Conversely, at high osmolarity, elevated OmpR-P represses *ompF* and activates transcription of *ompC* [25,26,27]. Upon phosphorylation by EnvZ in a high osmolarity environment, OmpR also directly induces transcription of both small RNAs OmrA and OmrB. Notably, OmrB can be activated at lower OmpR-P levels than OmrA, which is likely explained by its higher basal expression [18]. OmrA/B transcription can also be modulated by unphosphorylated OmpR. Overproduction of this form of OmpR still enhanced *omrA*/*B* expression, suggesting that both OmpR forms may interact with their promoters [28].

Further research showed that OmrA/B sRNAs negatively autoregulate their transcription by directly repressing the *ompR-envZ* operon that encodes their transcriptional activator. This creates a negative feedback loop in which OmpR activates *omrA*/*B* transcription, and OmrA/B repress *ompR-envZ* mRNA, thereby limiting their own expression. Unlike other OmpR targets such as *ompC* and *ompF*, OmrA/B expression is sensitive to the overall OmpR protein concentration rather than exclusively to OmpR-P levels. Experimental evidence shows that deletion of *omrA*/*B* increases transcription from the *ompR-envZ* promoter, while OmrA/B overexpression represses their promoter without significantly affecting *ompC* or *ompF* expression. Thus, OmrA/B specifically fine-tune their own expression without disturbing the balance of the major porins [18,28,29].

### 2.2. Alternative Sigma Factor by RpoS (σ^S^)

RpoS, also known as σ^S^, is an alternative sigma factor in *E. coli* (and related bacteria) that plays a central role in orchestrating the general stress response. It enables survival under diverse stress conditions such as stationary-phase entry, nutrient deprivation, oxidative stress, acidic environments, and osmotic stress. RpoS accomplishes this by directing RNA polymerase to transcribe a large set of stress-protective genes, the RpoS regulon, with over 300 promoters under its control [30].

In *Salmonella enterica*, OmrA/B are tightly integrated into regulatory networks that control virulence and stress adaptation. OmrA belongs to the group of sRNAs whose promoters show hallmarks of RpoS-dependent regulation, specifically containing conserved features such as a cytosine at the -13 position that distinguish them from promoters recognized by the housekeeping sigma factor RpoD [31].

OmrA/B expression are not regulated identically. In the Northern blot experiments, OmrA was detected in the wild type but its signal was lost or strongly reduced in the Δ*rpoS* strain, showing that transcription of *omrA* is increased in the presence of RpoS. By contrast, *omrB* is not significantly affected, indicating that it is controlled by other regulatory pathways rather than RpoS. This suggests that OmrA integrates input from RpoS together with other regulators such as OmpR, allowing it to remain active in stationary phase where it can downregulate a wide range of genes involved in processes like outer membrane protein synthesis, metabolism, motility, and biofilm formation. Whereas, OmrB is expressed at very low levels under the same stationary phase conditions, despite its similarity to OmrA and shared regulation by OmpR, demonstrating that the two sRNAs are differentially regulated in late stationary phase. This regulatory difference may help explain conservation of both sRNAs: OmrA, tied directly to σ^S^, under nutrient limitation or stationary-phase conditions, while OmrB responds independently to other signals. Such diversification provides flexibility: although OmrA/B share targets, their distinct expression controls may enable them to act in different contexts [32].

Another study identified a clear RpoS-binding site upstream of *omrA*, but not *omrB* in *E. coli*. This further supports that *omrA* is directly regulated by σ^S^, while *omrB* is not. Analyses showed that inactivation of *rpoS* almost abolished *omrA* transcription, while *omrB* expression was unaffected and even slightly increased in the *rpoS* mutant background. Thus, *omrA* is almost completely σ^S^-dependent, whereas *omrB* is σ^S^-independent. The basis for this regulatory divergence lies in their promoter sequences. The *omrA* promoter contains a cytosine residue at position -12 within its -10 region, a determinant that favors recognition and promoter opening by σ^S^ but not by the housekeeping σ^70^. In contrast, the *omrB* promoter carries the canonical -12T, which is optimal for σ^70^ recognition, explaining its independence from σ^S^. Mutagenesis experiments confirmed this: substituting the -12C of the *omrA* promoter with -12T drastically increased promoter strength and abolished its dependence on σ^S^. These results demonstrate that the -12C is a key specificity element for σ^S^ regulation of *omrA*. Thus, OmrA/B have diverged in promoter architecture and regulatory logic: *omrA* is integrated into the σ^S^ regulon as part of the stationary-phase stress response, while *omrB* is expressed independently, likely responding to distinct environmental signals [17].

## 3. Target Specificity of OmrA/B

**Figure 1:**
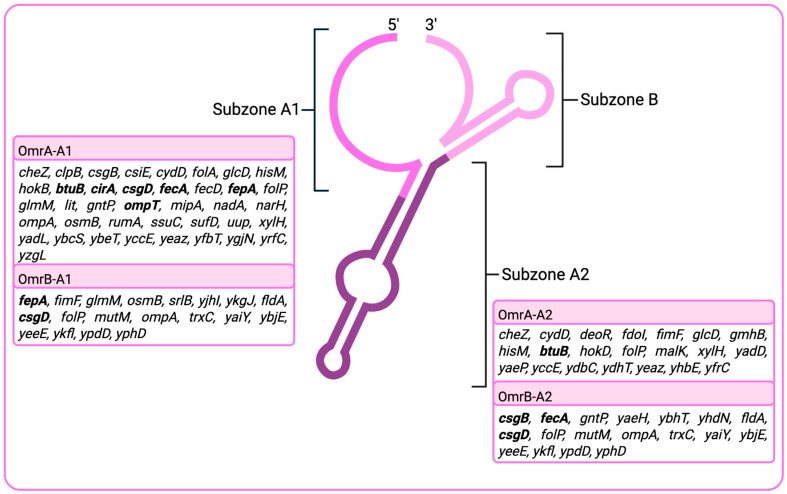
Interaction subzones located in the predicted secondary structure of the OmrA/B, based on Tello et al. (2018) [33]. Boxes indicate individual mRNA targets, with interaction subzones mapped relative to the sRNA-binding sites determined in the study. Genes shown in bold correspond to those highlighted in this review article as experimentally validated OmrA/OmrB targets. Created in BioRender [https://BioRender.com/hfis3cu (accessed on 20 September 2025)].

In *E. coli*, OmrA/B functions through a modular interaction structure with its diverse mRNA targets. Identified as an sRNA with an unusually large interaction set, OmrA regulates approximately 50 mRNAs, with distinct sequence subzones specialized for binding different transcript regions. The study by Tello et al. (2018) [33] divided the OmrA sequence into three subzones, A1, A2 and B. Subzone A1 (Figure 1), located near the 5′ end, interacts with 39 mRNAs, ~89% of which occur within the middle of coding sequences (CDS-M). In contrast, subzone A2, positioned closer to the 3′ end, associates with 21 mRNAs, ~60% of which map to 5′ untranslated regions (5′ UTRs). Furthermore, it was shown that none of the selected mRNA targets interacted with subzone B. This division suggests a modular specialization: A1 primarily engages coding regions, whereas A2 preferentially targets upstream regulatory regions, enabling OmrA to fine-tune translation at multiple stages. Conserved sequence motifs were also identified in target mRNAs. A1 recognizes a six-base motif (CUCUGG) present in 21 of its 39 targets, including *gntP*, *ompT*, *fecA*, *fepA*, and *glmM*. Similarly, A2 binds a six-base consensus motif (GCGUAC) found in all 20 of its targets, including *malK*, *btuB*, *glcD*, and *fimF*. Only *xylH* contained motifs for both subzones, indicating dual regulation through independent interaction sites. These conserved binding motifs demonstrate that OmrA acts not through random interactions, but by recognizing recurrent sequence patterns across families of mRNAs. Notably, most conserved interactions occur within coding sequences rather than untranslated regions, underscoring the CDS as a critical platform for sRNA-mediated regulation. OmrB demonstrates a similar pattern, there were 19 identified mRNA targets for subzone A1 and 18 for A2 subzone [33].

## 4. Iron Homeostasis and Regulation of Siderophore Uptake

OmrA/B may play a key role in relieving pressure on the machinery responsible for targeting and properly inserting proteins into the outer membrane (OM). Several essential proteins involved in this process have been identified [18,34], and overproduction of porins is known to trigger the “membrane stress response” [35]. Physiologically, the genes they regulate, including iron acquisition systems, fimbriae, adhesins, proteases, and therefore also crucial for pathogen-host interactions [29].

CirA, FecA, and FepA are outer membrane receptors specialized for the uptake of iron–siderophore complexes, and their genes are part of the Fur regulon. The Fur-Fe^2+^ protein represses expression of the iron regulon, including *cirA*, *fecA*, and *fepA*. Under low-iron conditions, when Fur is in its inactive state, expression of these iron-uptake genes is induced [36,37]. Enterobactin is the prototypical and one of the most efficient siderophores. The iron–enterobactin complex (Fe-Ent) is recognized by the outer membrane receptor FepA, transported across the outer membrane by a TonB-dependent mechanism, and subsequently translocated across the inner membrane via the FepBCG ATP-binding cassette system. Once inside the cell, the Fes esterase cleaves the enterobactin molecule to release iron, ensuring effective acquisition. Together, these features underscore the central role of enterobactin and FepA in microbial iron homeostasis [38].

*Yersinia enterocolitica* carries a functional OmrA homolog with conserved sequence and structural features similar to those of *E. coli*, while OmrB is absent, consistent with genomic searches showing no second homolog. In *Y. enterocolitica*, OmrA plays a key role as a post-transcriptional regulator of iron homeostasis, acting together with OmpR. It downregulates expression of *fecA* and *fepA*. Computational predictions indicated that OmrA base-pairs with regions in the early coding sequences of these mRNAs rather than in the 5′ UTR. Deletion of *omrA* resulted in significantly increased transcript levels of both *fecA* and *fepA*, with effects particularly increased under iron-limited conditions. These findings demonstrate that OmrA normally functions to silence these iron uptake systems [39].

In *Y. enterocolitica*, OmrA is also under the transcriptional control of OmpR, a component of the EnvZ/OmpR two-component system. OmpR binds directly to the *omrA* promoter, and *omrA* expression was strongly reduced in a Δ*ompR* mutant. Environmental factors such as osmolarity, pH, and temperature further influenced *omrA* expression, showing that OmrA connects environmental sensing to iron homeostasis. OmrA also responds back to regulate *ompR-envZ* itself. Predicted base-pairing between OmrA and *ompR-envZ* mRNA overlaps the ribosome-binding site, and experiments confirmed that OmrA reduces *ompR-envZ* transcript. This establishes a regulatory loop: OmpR activates OmrA transcription, while OmrA represses OmpR post-transcriptionally. Together, these findings establish OmrA as a key regulator in *Y. enterocolitica* iron homeostasis, acting post-transcriptionally to silence siderophore transporter genes while also engaging in feedback regulation with OmpR. RNA chaperone Hfq and the small RNA OmrA cooperate to regulate key pathways involved in iron homeostasis, and outer membrane composition. Our recent work has shown that Hfq silences several iron-related genes, including *fur*, *fecA*, and *fepA*, by reducing protein synthesis, while OmrA represses the iron uptake genes *fecA* and *fepA*. This regulation ensures tight control of iron acquisition, which is critical for bacterial adaptation and virulence in host environments. This suggests that Hfq participates in this process, likely stabilizing OmrA and promoting its base-pairing with target transcripts [39].

**Figure 2:**
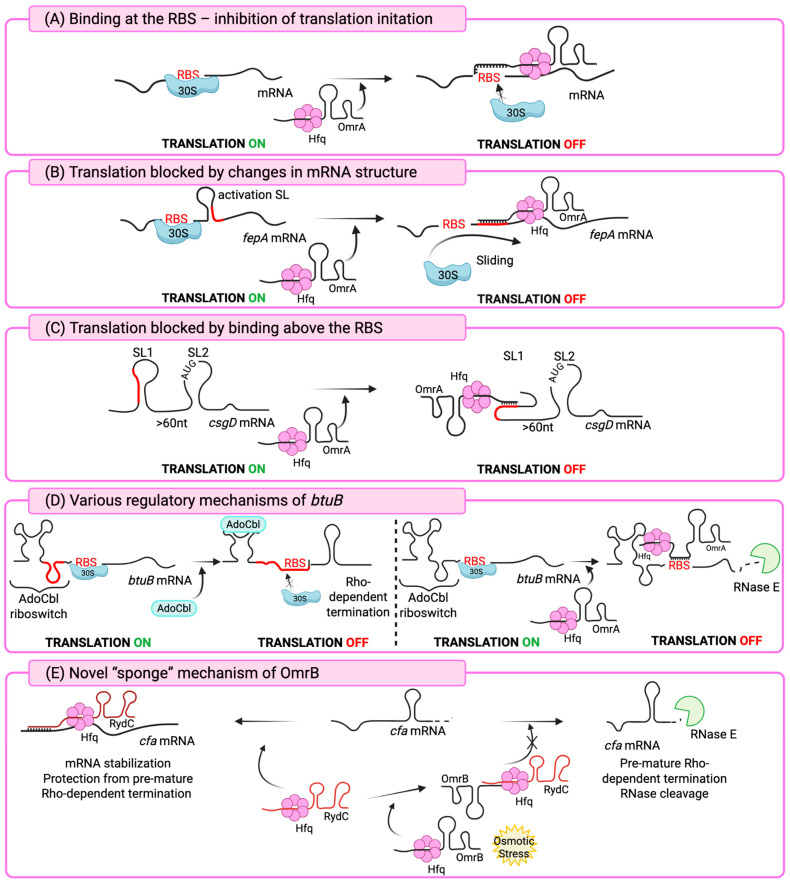
Schematic representation of the regulatory mechanisms of sRNAs OmrA/B. Different modes of OmrA/OmrB regulatory activity are shown: inhibition of translation via RBS binding (**A**), alteration of mRNA structures (**B**,**C**), *cis*-regulation of *btuB* through riboswitch and RBS binding by OmrA (**D**), and the novel “sponge” function of OmrB (**E**). See text for details. Created in BioRender [https://BioRender.com/7gccqgi (accessed on 20 September 2025)].

A particularly interesting example of OmrA/B regulatory function is the unique mechanism by which they control expression of the *fepA* gene in *E. coli*. Unlike the classical mode of repression, in which sRNAs base-pair with the translation initiation region to block ribosome access (Figure 2A), both OmrA and OmrB instead use their conserved 5′ end sequences to directly pair with a short region of the *fepA* coding sequence (Figure 2B), located at positions +14 to +22 downstream of the start codon. The *fepA* transcript contains an activating stem-loop (SL) structure around nucleotide +20 of the open reading frame, which normally enhances translation initiation by serving as a “starting block” for the ribosome. Since OmrA/B pairing with *fepA* does not overlap the ribosome-binding site, repression occurs through disruption of this stem-loop, thereby preventing efficient ribosome binding. This represents a novel model of sRNA-mediated gene regulation. The study shows that translation activation by SL is not limited to *fepA* but can also occur in other mRNAs, exemplified by *bamA*, which encodes an essential outer membrane protein assembly factor [40].

Experimental evidence from toeprinting assays confirmed that OmrA and OmrB binding to the stem-loop interferes with formation of the translation initiation complex, blocking ribosome access to *fepA* mRNA. The requirement for complementary pairing was demonstrated through mutational analysis: disrupting base-pairing sites in either OmrA/B or *fepA* abolished repression, while compensatory mutations restoring complementarity re-established regulation. These results underscore that direct base-pairing between OmrA/B and *fepA* is essential for blocking translation. Importantly, repression persisted even in the absence of RNase E. Using an RNase E mutant strain with reduced activity, the authors showed that *fepA* repression still occurred, indicating that OmrA/B act primarily by blocking translation rather than by initiating mRNA cleavage. Although both sRNAs use the same complementary 5′ sequence to pair with the +14 to +22 region of mRNA *fepA*, the study reported differences in efficiency. OmrA repressed translation of *fepA* more strongly, likely due to forming a more stable interaction with this region or engaging more effectively with the surrounding RNA structure. OmrB also repressed *fepA* expression but less efficiently, making its effect weaker and more condition-dependent. This functional difference helps explain the retention of both sRNAs in *E. coli*: OmrA may act as the primary repressor of *fepA*, while OmrB plays a secondary role, providing fine-tuning or redundancy. This post-transcriptional regulation is integrated into the broader EnvZ/OmpR regulatory network, which induces OmrA/B expression in response to various stress environments. By repressing *fepA*, OmrA/B help limit iron uptake under stress conditions, acting in cooperation with transcriptional regulators such as Fur to coordinate iron homeostasis. OmrA/B therefore help restrict not only the production of FepA but also other iron siderophore receptors, such as FecA and CirA, in conditions where siderophore-mediated iron uptake is unnecessary or could even be toxic. Moreover, downregulation of immunogenic outer membrane proteins such as FepA, which has been shown to induce protective antibody responses in mice, may also support bacterial evasion of host immune defenses [40,41].

## 5. Regulation of Motility and Biofilm Formation

Enterobacteria, including *E. coli* and *S. enterica*, adjust their lifestyle according to environmental cues, altering between a sessile biofilm state and a motile, flagellated form. These two states are generally viewed as incompatible, and this balance is maintained through complex transcriptional networks that promote one lifestyle while simultaneously repressing the other [42,43].

FlhDC is the master regulator of flagellar biosynthesis in motile bacteria such as *E. coli* and *S. enterica*. The *flhD* and *flhC* gene products form a heterohexameric FlhD_4_FlhC_2_ complex that binds upstream of flagellar operons, bends DNA, recruits RNA polymerase, and activates basal body gene transcription, initiating the flagellar assembly hierarchy [44]. FlhDC drives σ^70^-dependent transcription of class II genes [45], which build the hook-basal body (HBB). Class III gene expression requires σ^28^ (FliA), produced from class II, but initially inhibited by FlgM [46,47]. The *flhDC* operon, transcribed by σ^70^ RNA polymerase, is tightly regulated: activated by CRP and H-NS, and repressed by LrhA, HdfR, and two-component systems such as EnvZ/OmpR [44].

The *flhDC* mRNA contains a conserved 198-nucleotide 5′ untranslated region (5′ UTR), and the global regulator CsrA has been shown to bind this leader sequence, enhancing translation [48]. Analyses suggest that OmrA forms extensive predicted base-pairing with the *flhDC* leader sequence, whereas OmrB exhibits weaker complementarity, consistent with its reduced ability to regulate *flhDC* expression. In motility assays, OmrA overexpression strongly reduced motility, nearly eliminating it, while OmrB produced a milder but still significant reduction. At the molecular level, OmrA and OmrB reduced expression of a translational *flhD′–′lacZ* fusion. Mutations introduced into OmrA and OmrB disrupted their ability to regulate this fusion, whereas compensatory mutations in the *flhD* leader restored repression. This demonstrated that regulation occurs through direct base-pairing with the 5′ UTR of *flhDC* mRNA, near the ribosome-binding site, thereby likely blocking ribosome access and translation initiation (Figure 2A). It was also suggested that alternative interaction patterns may exist, since disrupting a single predicted pairing site did not fully abolish repression, consistent with the extensive potential complementarity between the sRNAs and the *flhDC* leader sequence [49].

OmrA/B also directly repress synthesis of the anti-sigma factor FlgM, which normally inhibits the sigma factor σ^28^ (FliA). Both sRNAs bind through complementary base-pairing to the early coding region of *flgM* mRNA, close to the start codon, thereby blocking ribosome binding. Computational predictions (IntaRNA) identified base-pairing interactions between the conserved 5′ seed sequences of OmrA and OmrB and the early coding region of *flgM*. Disrupting these base-pairing sites reduced OmrA/B-mediated repression of *flgM*, while compensatory mutations that restored complementarity re-established regulation. OmrA/B prevent formation of the 30S initiation complex on *flgM* mRNA, and both sRNAs, particularly OmrA, strongly decrease FlgM protein synthesis. OmrA/B lower FlgM levels, releasing σ^28^ to activate class III flagellar genes like *fliC*, which encodes flagellin. Deletion of *flgM* increases class III expression without affecting class II, and OmrA/B-mediated FlgM repression produces a similar, though weaker effect. Engineered mutants showed that OmrA’s control of *flhDC* and *flgM* can be separated, and that targeting *flgM* alone is enough to increase *fliC* expression [50]. Hfq plays a central role in enabling OmrA/B to regulate the *flgM* gene expression in *Enterobacterales*. Hfq stabilizes OmrA/B against ribonuclease degradation and promotes their imperfect base-pairing with the *flgM* transcript, ensuring efficient translational inhibition. Experimental evidence shows that deletion of *hfq* abolishes OmrA/B repression of *flgM*, even if the sRNAs are expressed, demonstrating that Hfq is essential for the interaction. In this regulatory circuit, Hfq is not only a stabilizer but also a catalyst of post-transcriptional control, ensuring that OmrA/B repression of *flgM* effectively links sRNA regulation to flagellar gene expression and motility outcomes [50].

CsgD (encoded by *csgD*) is a transcriptional regulator of the LuxR family that functions as the central “master regulator” of biofilm formation in *E. coli*. It activates expression of the curli genes, particularly the *csgBAC* operon, and also promotes cellulose synthesis, another major component of the biofilm extracellular matrix [51]. By positively regulating *csgBAC* operon, CsgD drives curli fiber assembly [52]. Multiple environmental stimuli, including temperature, osmolarity, nutrient availability, and oxygen levels, modulate curli gene expression [53].

Both OmrA and OmrB in *E. coli* function in parallel to repress expression of the transcriptional regulator CsgD. Either sRNA alone is sufficient for repression, and overexpression of OmrA/B abolishes curli fiber formation and reduces cellulose production, demonstrating their broad influence on extracellular matrix components and biofilm traits. OmrA/B repress *csgD* by direct antisense base-pairing with the 5′ untranslated region of the mRNA, as with previously described targets. However, their binding site lies far upstream of the ribosome-binding site (RBS), distinguishing this regulation from most antisense RNAs, which act by directly blocking ribosomal access [54].

The *csgD* leader is unusually long (~150 nucleotides) and contains two structured modules, termed SL1 (stem-loop 1) and SL2 (stem-loop 2). OmrA/B base-pair with ~18–19 nucleotides in the SL1 region, located ~60–80 bases upstream of the start codon (Figure 2C). This interaction occurs in a bulge that makes the target accessible, and binding remodels the SL1 structure by partially opening its stem. SL2, located closer to the Shine–Dalgarno sequence, normally sequesters it in a hairpin, reducing translation efficiency. Mutational analyses demonstrated that SL1 is essential for OmrA/B regulation, while SL2 primarily tunes basal expression. Despite the distant binding site, OmrA/B pairing prevents initiating ribosome binding at the translation start region, making this an unusual example of long-distance sRNA-mediated repression. Unlike other sRNAs such as McaS, RprA, and GcvB, OmrA/B do not promote endonucleolytic cleavage or degradation of *csgD* mRNA. Instead, they inhibit translation in a cleavage-independent manner by restricting ribosomal access. Functionally, repression of *csgD* by OmrA/B consequently suppresses the *csgBA* operon, thereby preventing biofilm-associated traits under conditions that favor motility. OmrA/B may also act as “scavengers,” eliminating misregulated *csgD* transcripts and fine-tuning cell surface remodeling and group behavior in bacterial populations [54].

The RNA chaperone Hfq is indispensable in the regulatory pathway of controlling *csgD*. Deletion of the *hfq* gene leads to drastically reduced levels of *csgD* mRNA and of OmrA/B themselves, showing that Hfq is required for the stability of both the sRNAs and their target mRNA even before any sRNA-mediated inhibition occurs. Moreover, when *hfq* is deleted, sRNA-dependent repression is effectively abolished, even when OmrA/B are overexpressed [54]. Hfq promotes pairing between sRNAs and mRNAs, and regulatory models suggest that an Hfq-binding site within the *csgD* leader not only stabilizes sRNA binding but may also recruit RNase E, promoting degradation of the *csgD* transcript [55,56]. This places them alongside sRNAs such as RydC and RybB, which inhibit translation by base-pairing near or upstream of the RBS without triggering transcript degradation, representing a distinctive mode of post-transcriptional regulation [54,55].

DgcM is one of several GGDEF-domain diguanylate cyclases in *E. coli* that generate c-di-GMP, a key regulator of biofilms, motility, and the transition between sessile and motile lifestyles [57,58]. Within a local signaling module, DgcM interacts with PdeR and MlrA to control *csgD* and curli expression [59]. OmrA/B repress DgcM by base-pairing near or overlapping its ribosome-binding site. Mutational analysis confirmed this interaction: disrupting pairing eliminated regulation, and compensatory mutations restored OmrB’s but not OmrA’s repression, implying that OmrA may additionally bind to a second, less functional site. In strains with *csgD* and *ompR* mutation, OmrA, but not OmrB, still repressed biofilm formation, likely via *dgcM*. Two adjacent binding sites were identified on the *dgcM* mRNA. Both OmrA and OmrB recognize site 1, but site 2 is bound differently: OmrA can extend its binding into an additional region that OmrB cannot reach. The differential ability to engage site 2 is key to explaining why OmrA retains repression in the *csgD*/*ompR* mutant backgrounds while OmrB does not. Moreover, gel-shift assays demonstrated that OmrA binds more efficiently than OmrB and can simultaneously occupy both sites on a single mRNA molecule, suggesting cooperative or extended base-pairing that strengthens binding and regulatory efficacy. Repression of *dgcM* translation critically depends on the RNA chaperone Hfq. Unlike its classical role as a passive matchmaker, Hfq plays an active remodeling role in this case. Specifically, Hfq’s distal face is required to unfold a structured RNA hairpin within the *dgcM* 5′ region, exposing the otherwise inaccessible OmrA/B binding site. A distal-face mutant abolishes regulation in vitro and in vivo, while proximal/rim mutations have little effect. This mechanism demonstrates that Hfq is not merely a stabilizer of OmrA/B but under certain circumstances is essential for granting them access to their target sequence, highlighting its dual role as both RNA chaperone and RNA remodeler [60].

RelA is a stringent response regulator in *E. coli* and many other bacteria that produces the signaling molecules ppGpp and pppGpp, which are central to the stringent response, which is triggered during stress [61,62]. RelA recognizes GGAG or GGAGA motifs [63] and facilitates the regulation of mRNAs carrying such motifs by sRNAs that themselves lack GGAG. DgcT, formerly known as YcdT, is a diguanylate cyclase, which is associated with repression of swimming motility in *E. coli* [64]. The predicted binding site of OmrB on the *dgcT* mRNA spans nucleotides −12 to +2 relative to the AUG start codon, while the GGAG site is located much further downstream, at +294 nt relative to AUG. Thus, the OmrB binding site does not overlap with the RelA-recognized GGAG motif. It was proved that in the wild-type strain RelA facilitated regulation of *dgcT′-′lacZ* fusions by OmrB, regulation was lost in the Δ*relA* mutant and in the RelA:C289Y mutant (defective in GGAG recognition), which confirms that RelA protein is required for said regulation. (p)ppGpp synthesis and RNA binding are mutually exclusive activities of RelA. Furthermore, amino acid starvation is an inducer of (p)ppGpp synthesis and reduces RelA’s ability to promote base-pairing regulation. Amino acid starvation decreased *dgcT* fusion protein expression in *relA* mutants relative to non-starved controls, under conditions of OmrB overexpression, which indicates that *dgcT* regulation by OmrB depends specifically on RelA binding to the GGAG motif. Overall, the findings suggest that RelA promotes sRNA-mRNA pairing by binding to GGAG-containing mRNAs and, much like in RelA-sRNA-Hfq interactions, stabilizes otherwise unstable mRNA-Hfq monomer complexes, enabling their assembly into an Hfq hexamer that supports efficient sRNA-mRNA duplex formation [63,65].

The article by Zeng and Sundin (2014) [66] reports that the single small RNA named OmrAB acts as a regulator in *Erwinia amylovora*, the causative agent of fire blight disease in apple and pear trees. The group identified OmrAB among the sRNAs with important roles during infection. Functional assays showed that OmrAB promotes motility while repressing production of amylovoran, an exopolysaccharide that is a major component of biofilms [67]. These activities suggest that OmrAB coordinates the pathogen’s transition between infection stages. Motility is important at early stages for bacterial entry into plant tissues, while amylovoran and biofilm formation are critical at later stages for establishing systemic infection. Notably, this regulatory pattern differs from *E. coli*, where OmrA/B act as negative regulators of motility. In *E. amylovora*, by contrast, OmrAB functions as a positive regulator of swimming motility, indicating that while their role as motility regulators is conserved, the precise mechanisms and outcomes are species-specific [66]. This finding was later re-examined, and functional experiments revealed that OmrAB acts as a positive regulator of flagellar motility. The authors proposed that OmrA/B must also target additional motility-related mRNAs (beyond *flhDC*). Candidate targets include FlgD (a flagellar assembly protein), a methyl-accepting chemotaxis protein, and potentially the global regulator H-NS. Moreover, when *E. amylovora omrAB* was expressed in *E. coli*, it behaved like its *E. coli* counterpart and repressed motility, which underscores that the regulatory pathways differ between the two bacteria Assays confirmed that strains with Δ*omrAB* mutation exhibited reduction in swarming motility, and it was restored after introduction of *omrAB* with a native promoter on a complementation plasmid [68].

OmrAB influences flagellar regulation by acting on the *flhDC* transcript at the post-transcriptional level. To test this, Schachterle et al. (2019) [68] introduced an *flhDC* translational fusion into *E. coli* strains engineered to produce *E. amylovora* OmrAB. Reporter signal was strongly reduced, indicating that OmrAB directly repressed translation of *flhDC*. This regulatory effect was specific to translation rather than transcript stability, as the half-life of *flhDC* mRNA was unchanged in the Δ*omrAB* mutant compared to wild-type or other sRNA mutants. Since OmrA and OmrB are known in *E. coli* to directly bind the *flhDC* mRNA, the conservation of these interactions was evaluated in *E. amylovora*. Sequence alignments across *Enterobacterales* showed moderate conservation of OmrAB itself and of the corresponding OmrAB-interacting region in the *flhDC* 5′ UTR, supporting the idea that this regulatory interaction is evolutionarily preserved. Taken together, these findings demonstrate that OmrAB acts as a conserved post-transcriptional repressor of *flhDC* in *E. amylovora*, influencing flagellar regulation by reducing translational efficiency rather than altering mRNA stability [68].

## 6. Virulence Regulation

Beyond their roles in metabolic regulation and cell envelope remodeling, OmrA and OmrB also modulate virulence-related phenotypes in diverse bacterial pathogens. In *Klebsiella pneumoniae*, the capsule (CPS) is a critical virulence factor that protects the bacterium from host immunity by preventing phagocytosis and complement system activation. Hypervirulent strains (hvKp) frequently produce unusually large amounts of capsule, resulting in a hypermucoviscous (HMV) phenotype. This trait is associated with enhanced survival in host tissues and increased transmission [69]. KvrA, a LysR-type transcriptional regulator, positively regulates capsule gene expression. Deletion of *kvrA* results in decreased capsule production and reduced hypermucoviscosity, leading to attenuated virulence in animal infection models. KvrA likely functions by directly or indirectly activating transcription of capsule biosynthesis genes and possibly interacting with other global regulators. These findings place KvrA as a central regulator linking capsule synthesis to the overall virulence of *K. pneumoniae* [70].

OmrB was found to base-pair with *kvrA* mRNA, and GFP reporter assays confirmed that OmrB binding suppressed *kvrA* translation. OmrB overexpression also decreased KvrA protein levels under different growth conditions. Mutational analysis, in which compensatory mutations were introduced into both *omrB* and *kvrA*, demonstrated that repression depends on direct base-pairing between the two RNAs. Functionally, OmrB overexpression reduced both capsule production and HMV phenotype, as shown by uronic acid quantification and sedimentation assays. However, deletion of *omrA/B* did not result in substantial changes in capsule production or HMV compared to the wild type, even under conditions known to induce OmrB expression in *E. coli* (nutrient broth with 20% sucrose). This suggests the presence of additional sRNAs that also contribute to capsule regulation. Like OmrB, OmrA overexpression also decreased capsule production and HMV, indicating that both sRNAs can act as negative regulators of these key virulence traits. Interestingly, OmrB’s role in capsule regulation appears complex: while it repressed *kvrA* translation, capsule phenotypes in different experimental setups did not always perfectly align with *kvrA* expression inhibition. This suggests that OmrB may influence capsule regulation through additional targets or indirect pathways. In conclusion, OmrA/B act as repressors of capsule production and hypermucoviscosity in K. pneumoniae. OmrB directly downregulates *kvrA* through base-pairing and OmrA produces similar phenotypes while overexpressed [71].

Enteropathogenic *E. coli* (EPEC) and enterohemorrhagic *E. coli* (EHEC) are closely related but differ in disease patterns and host impact. EPEC mainly affects children under two in developing countries, causing severe watery diarrhea. EHEC, more common in industrialized nations, affects both children and adults, leading to bloody or non-bloody diarrhea and sometimes hemolytic uremic syndrome (HUS)—a serious complication that can cause lasting kidney damage and require dialysis or transplantation. Unlike EPEC, EHEC produces Shiga toxins that damage renal endothelial cells and trigger HUS [72]. The LEE is a pathogenicity island (PAI) of ~35–40 kilobases present in EPEC and EHEC, and some related pathogens (like *Citrobacter rodentium*). These bacteria rely on a type III secretion system (T3SS) to translocate effector proteins from the bacterial cytosol into host cells [73]. LEE is essential for “attaching-and-effacing” (A/E) lesions formation. It contains ~41 genes, organized in operons named LEE1-LEE7 that encode a T3SS, its chaperones, secreted effectors, adhesins, and regulators [74,75]. LEE1, LEE2, and LEE3 contain the *esc* and *sep* genes, which encode the major components of the T3SS, while LEE1 also carries the *ler* gene encoding Ler, a DNA-binding protein that functions as the primary transcriptional regulator of the LEE region and modulates expression of the other LEE operons [76]. Attaching and effacing lesions are signature features of infection, characterized by tight bacterial adhesion to the host plasma membrane, destruction of enterocyte microvilli, and cytoskeletal rearrangements beneath the adherent bacteria [73].

OmrA/B are identified as important virulence factors in enteropathogenic *E. coli* (EPEC), since both sRNAs globally silence genes of the LEE. Overexpression of OmrA/B strongly reduced the protein levels of LEE-encoded effectors, including EspA (encodes a secreted filamentous protein that forms a filament connecting the bacterial T3SS to the host cell membrane), EspB (translocator protein, required for creating a pore in the host cell membrane), and Tir (principal effector that is translocated into the host cell and then inserted into the host plasma membrane, acting as a receptor for the bacterial outer membrane adhesin intimin) [77,78]. This correlated with a strong decrease in the mRNA abundance of LEE operons genes *espA* and *tir*, showing that repression occurred at the transcript level. Overall, deletion of *omrA*/*B* alone had little effect, but overexpression remarkably decreased LEE gene expression, indicating that these sRNAs act as effective repressors when present at high levels. Mechanistically, OmrA/B do not directly base-pair with LEE transcripts. Instead, they indirectly repress transcription of the LEE master regulator Ler, which normally activates all other LEE operons. Reporter fusion assays, under LEE1 promoter (which controls *ler*) showed that OmrA and OmrB reduce its expression but not by direct interaction with *ler* mRNA. Instead, the effect seems to be mediated by an unidentified transcription factor that binds within 200 base pairs upstream of the transcription start site of the LEE1 promoter. Since OmrA/B reduced *ler* expression without a base-pairing mechanism, the authors concluded that repression is indirect, likely mediated through another transcription factor or regulator that acts upstream of *ler* [78].

## 7. Riboswitch and Small RNA: Regulation of *btuB*

The *btuB* riboswitch is a cis-regulatory RNA structural element embedded in the 5′ UTR of the *btuB* mRNA, which encodes an outer membrane transporter responsible for importing vitamin B_12_ (cobalamin) into the cell. Its expression is regulated in response to intracellular levels of adenosyl-cobalamin (AdoCbl), the biologically active form of vitamin B_12_, through alterations in mRNA folding [79,80]. The riboswitch depends on a “kissing loop,” a specific interaction in which two RNA hairpin loops pair with each other. This interaction is essential both for ligand recognition and for stabilizing the RNA in its repressive conformation that prevents gene expression [81]. Importantly, folding is coordinated with transcriptional pausing by RNA polymerase, which allows the aptamer to adopt the correct structure while the mRNA is still being synthesized [79].

OmrA and OmrB both participate in repressing *btuB* expression in *E. coli*, but they do so with different efficiencies and by partially distinct mechanisms. As previously described, OmrA/B typically regulate their targets by base-pairing through a conserved sequence at their 5′ end. However, in the case of *btuB*, OmrA uses its central region, which is not conserved in OmrB, to recognize and pair with the mRNA. Sequence analyses revealed that this central portion of OmrA is complementary to the translation initiation region of *btuB*, overlapping the ribosome-binding site, which explains its strong repressive effect. By contrast, OmrB shows only partial complementarity to this region, and its ability to base-pair with *btuB* mRNA is much weaker. Thus, while OmrA can form extensive and stable interactions that effectively block translation, OmrB’s binding is less precise and less efficient, so it contributes only weakly and primarily in the absence of OmrA, only by interacting with 5′ UTR of *btuB* [82].

Experiments with *btuB′-′lacZ* fusions demonstrated that OmrA and OmrB regulate *btuB* primarily at the translational level, with OmrA exerting stronger effects than OmrB. Translational fusions carrying the *btuB* 5′ UTR and increasing lengths of the coding sequence (CDS) revealed that OmrA reduced expression by at least 50% when 81 nt of the CDS were present, and repression became much stronger as the CDS length increased. OmrB showed a similar trend but consistently lower efficiency, with maximal repression plateauing at about 50% for the longer constructs. In contrast, transcriptional *btuB::lacZ* fusions containing the same CDS regions, but fused to *lacZ* with its own initiation signals, were mostly unaffected by OmrA/B. These results indicate that OmrA/B do not strongly influence transcriptional output but act primarily at the translational level, with decreased mRNA levels likely being a downstream consequence of translational inhibition, most plausibly through RNase E-mediated cleavage of ribosome-free transcripts beyond nucleotide 210 of the *btuB* CDS. Mutational analyses confirmed that altering either the central region of OmrA or the complementary sequence in *btuB* strongly impaired repression. Introducing mutation in 5′UTR of OmrB abolished repression. These findings indicate that *btuB* is a preferential target of OmrA, with OmrB playing at most a minor or conditional role [82].

Physiological assays further confirmed OmrA’s dominance under acidic conditions, when its expression is induced by OmpR activation. In this environment, deletion of *omrA* caused a clear increase in *btuB* expression, while deletion of *omrB* alone had little effect. Only when both sRNAs were deleted repression was fully lost, highlighting OmrA’s primary role in controlling *btuB*. The study demonstrated that OmrA can regulate specific targets independently of OmrB, while OmrB’s contribution is probably context-dependent. This differential regulation likely explains why both sRNAs have been conserved in many enterobacteria despite their high sequence similarity. These findings establish *btuB* as an unusual case in which OmrA acts through its central region rather than its conserved 5′ end, making it a specific OmrA target. OmrB can contribute weakly, but only in the absence of OmrA. The regulation of *btuB* by OmrA strictly requires Hfq, but through a mechanism distinct from canonical OmrA/B targets like *ompR*. Using a translational fusion and *hfq* point mutants, it was shown that OmrA repressed *btuB* expression in the wild type, while OmrB had only a weak effect. This repression was lost in Δ*hfq*, as well as in the proximal face and rim of Hfq mutants, largely due to reduced OmrA/B accumulation in these strains. However, in the distal face mutant, OmrA and OmrB accumulated to high levels but no longer repressed *btuB*, while still strongly repressed *ompR*. This indicates that OmrA/B use the canonical 5′-end mechanism for *ompR* regulation but require Hfq binding at the distal face and recruitment to the mRNA for *btuB* regulation. Thus, OmrA-mediated repression of *btuB* relies on a Hfq-dependent mechanism distinct from its regulation of other targets [82].

This represents the first example of gene regulation in *E. coli* where a riboswitch and sRNAs act on the same mRNA through completely different molecular pathways. The riboswitch acts *in cis*, sensing adenosyl-cobalamin and altering its structure to block ribosome binding and trigger Rho-dependent transcription termination. In contrast, OmrA (and weakly OmrB) act *in trans* by base-pairing with *btuB* mRNA near the ribosome-binding site, which promotes degradosome activity and leads to mRNA decay. Thus, the riboswitch regulates *btuB* co-transcriptionally, while OmrA/B regulate it post-transcriptionally through targeted RNA degradation (Figure 2D) [82].

## 8. Novel “Sponge” Mechanism of OmrB

Regulation of the *cfa* gene expression, which encodes cyclopropane fatty acid (CFA) synthase, involves both transcriptional and post-transcriptional mechanisms. At the transcriptional level, RpoS plays a key role: osmotic stress activates RpoS, which promotes *cfa* transcription from an RpoS-dependent promoter. This ensures CFA synthase production under stress, enabling modification of membrane lipids for increased resilience [83,84]. Beyond transcription, sRNA RydC fine-tunes *cfa* expression post-transcriptionally by binding to its mRNA, protecting it from premature Rho-dependent termination and stabilizing the transcript [85]. OmrB was shown to have a novel role as a negative regulator of RydC. When OmrB was expressed in a Δ*yieP* mutant, an *E. coli* strain lacking *yieP*, which encodes a minor global transcriptional regulator [86], *cfa* expression was decreased. YieP normally represses *rydC*, so its deletion results in increased RydC levels, creating a background where OmrB’s repressive effect on *cfa* could be observed. This repression disappeared when *rydC* was deleted, demonstrating that OmrB does not act directly on *cfa* mRNA but instead interferes with RydC itself [87].

Experimental evidence supports a “sponge” model in which OmrB base-pairs with RydC, preventing it from activating *cfa* (Figure 2E). Mutational analysis confirmed this: introducing a point mutation into OmrB that weakened its complementarity to RydC eliminated its ability to repress *cfa*. These findings indicate that OmrB regulates *cfa* indirectly by controlling the availability and activity of RydC. Physiologically, this mechanism provides an additional layer of control over *cfa* expression during stress. Because OmrB is induced by regulators such as EnvZ/OmpR in response to environmental cues, it can fine-tune the balance between RydC-mediated activation and RpoS-driven transcription of *cfa*. This expands OmrB’s functional repertoire, showing that it can regulate activity of other sRNAs as well [87].

## 9. OmrA/B as Potential Synthetic RNAs

Synthetic small RNAs (sRNAs) serve as adaptable tools for post-transcriptional gene regulation in bacteria, enabling targeted repression through the combination of a customizable antisense seed region with a defined structural scaffold. In the modular plasmid system developed by Köbel et al. (2022) [88], this design principle is implemented through a Golden Gate based assembly strategy that allows rapid construction and benchmarking of synthetic sRNAs in *E. coli*. Their study focused on regulating *acrA*, a component of the AcrAB-TolC efflux pump implicated in β-lactam susceptibility, using oxacillin-based assays to evaluate regulatory efficiency [88].

In this system, the scaffold provides the RNA architecture that supports the function of the engineered sRNA, including stem-loops, an intrinsic terminator, and structural elements that facilitate interaction with the RNA chaperone Hfq, which stabilizes sRNAs and promotes sRNA-mRNA pairing. While the seed sequence determines target specificity, the scaffold dictates whether the seed is presented in an accessible conformation and whether the chaperone can effectively associate with the synthetic construct. These features are essential for achieving robust repression in vivo. The OmrB scaffold, tested alongside RybB, MicA, and MicF, retains natural structural motifs that support stability and Hfq-dependent activity, making it a relevant candidate for synthetic designs [88].

A central question in the study was whether validated antisense seed sequences maintain their regulatory potential when transplanted onto different scaffolds. To address this, the authors evaluated the widely used s8 seed, which originates from the 5′ target-recognition region of the outer membrane protein regulating sRNA RybB and was previously shown to act as an effective antisense module [89]. When fused to the native RybB scaffold (RybB-s8), the construct produced strong repression of *acrA* and a pronounced susceptibility phenotype in the oxacillin assays performed in their study. In contrast, when the same seed was fused to the OmrB scaffold (OmrB-s8), the regulatory effect was substantially weaker: only a slight inhibitory phenotype was detectable in their oxacillin plating assay, and overall activity was clearly reduced compared with RybB-s8. Importantly the functional data with RNAfold and Mfold predictions showing that the s8 seed becomes largely sequestered within a stable 5′ stem-loop in the OmrB context, provides an explanation for the reduced activity by indicating that the seed is likely less accessible for base-pairing with the *acrA* mRNA.

These scaffold-dependent differences are consistent with broader principles emerging from recent synthetic sRNA design research. A high-throughput library analysis demonstrated that scaffold architecture strongly influences several aspects of synthetic sRNA behavior, including seed-region accessibility, Hfq dependence, RNase E processing, and overall regulatory output, which highlights that seed sequences optimized in one backbone do not necessarily retain full activity in another. Their findings reinforce that scaffold-specific structural constraints must be considered when designing synthetic sRNAs [90].

Taken together, these results show that OmrB can function as a synthetic scaffold, but its regulatory efficacy is highly sensitive to structural compatibility between the scaffold’s 5′ conformation and the inserted seed. The weak activity of OmrB-s8 in the study illustrates that ensuring appropriate seed exposure is a critical design criterion. More generally, the combined insights emphasize that effective synthetic sRNA engineering requires integrating modular design with careful assessment of RNA folding, scaffold-specific structural features, and protein interactions to achieve predictable regulatory performance.

## 10. Conclusions

OmrA/B represent a typical example of how small RNAs integrate environmental sensing, stress adaptation, and virulence regulation within *Enterobacterales*. Emerging from an ancestral gene duplication, they have retained highly conserved terminal regions that act as structural domains for base-pairing with a wide range of target transcripts (Table 1), while their divergent central regions enable functional asymmetry. Their integration into the EnvZ/OmpR regulon ensures that their expression is responsive to environmental changes such as osmolarity and temperature, while feedback repression of *ompR-envZ* further integrates them within global signaling loops. By controlling outer membrane proteins, siderophore receptors, and envelope-associated enzymes, OmrA/B fine-tune membrane permeability and nutrient acquisition, functions that are essential for both survival in fluctuating niches and pathogenic success in host environments.

The regulatory scope of OmrA/B extends well beyond nutrient uptake. Through repression of *flhDC*, *flgM*, *csgD*, and *dgcM*, they directly link motility to biofilm formation, balancing mutually opposing lifestyles. Their interaction with riboswitch-controlled genes such as *btuB* illustrates how *cis-* and *trans*-regulatory mechanisms can converge on the same transcript, providing multilayered control of key metabolic pathways. In pathogenic species, including *K. pneumoniae*, *E. amylovora*, and *S. enterica*, OmrA/B contribute to virulence by modulating capsule synthesis, motility, and stress responses, while their target repertoires are shaped by species-specific regulatory contexts. These examples underscore their evolutionary flexibility: while they preserve a conserved regulatory core, their physiological outcomes vary across different bacterial hosts.

Central to all of these activities is the RNA chaperone Hfq, which is indispensable for OmrA/B stability, base-pairing efficiency, and access to structured target regions. In some cases, such as regulation of *dgcM* or *csgD*, Hfq acts not only as a stabilizer but also as an RNA remodeler, actively reshaping target structures to permit effective pairing. In this way, Hfq amplifies the regulatory reach of OmrA/B, ensuring that their imperfect base-pairing interactions can exert strong biological effects. Additional factors, such as RelA, further enhance their efficiency by modifying target RNA accessibility, thereby linking sRNA function to global stress pathways such as the stringent response.

One potential use of many bacterial sRNAs, including OmrA and OmrB, is to inspire the design of programmable antisense oligonucleotides (ASOs) for species-specific RNA antibiotics. Much like *trans*-acting sRNAs, ASOs silence gene expression through short, sequence-specific base pairing, typically targeting 5′ mRNA regions to block translation. Advances in modified nucleic acid chemistries and peptide-based delivery now allow ASOs to selectively inhibit essential genes across diverse bacteria, offering a level of precision far beyond broad-spectrum antibiotics. Importantly, mechanistic insights from natural sRNAs, including seed-based recognition, pairing within extended mRNA regions, and multiple modes of translational repression provide a conceptual framework for improving ASO design and specificity. As bacterial RNA biology continues to advance, sRNAs such as OmrA/B highlight how endogenous regulatory strategies can guide the development of next-generation RNA-based antimicrobials for species-level microbiome editing [91,92].

Together, OmrA/B illustrate the versatility of bacterial small RNAs as post-transcriptional regulators. They are not limited to the repression of single transcripts but act as central nodes that coordinate multiple cellular processes. Future research should continue to explore their species-specific functions, their interplay with global regulators, and their potential as scaffolds in synthetic biology. Ultimately, OmrA/B exemplify how sRNAs enable bacteria to fine-tune gene expression with remarkable precision, ensuring adaptability in both environmental and host-associated contexts.

## Figures and Tables

**Table 1:** Experimentally supported OmrA/B targets.

Target	Gene Function	Regulatory Mechanism	Regulated By	Species	Source
*ompR-envZ*	Two-component regulator of porin expression	Post-transcriptional repression, negative feedback loop	OmrA/B	*E. coli* *Y. enterocolitica*	[18,28,29,39]
*ompT*	Outer-membrane protease	Post-transcriptional repression	OmrA/B	*E. coli*	[18,29]
*cirA*	Siderophore receptor	Post-transcriptional repression	OmrA/B	*E. coli*	[18,29]
*fecA*	Ferric-citrate receptor	Post-transcriptional repression	OmrA/B	*E. coli*, *Y. enterocolitica*	[18,29,39,40]
*fepA*	Enterobactin receptor	Direct CDS pairing, blocks the SL of the *fepA* transcript → translation inhibition	OmrA/B	*E. coli*, *Y. enterocolitica*	[18,29,39,40]
*fimA*/ *fimI*	Fimbriae assembly	Post-transcriptional repression	OmrA/B	*E. coli*	[18]
*flu* *(Ag43)*	Adhesin	Post-transcriptional repression	OmrA/B	*E. coli*	[18]
*glmM*	Cell-wall synthesis	Post-transcriptional repression	OmrA/B	*E. coli*	[18]
*folP*	Folate biosynthesis	Post-transcriptional repression	OmrA/B	*E. coli*	[18]
*btuB*	Vitamin B12 receptor	RBS pairing → translation inhibition	OmrA strong, OmrB weak	*E. coli*	[82]
*flhDC*	Flagellar master regulator	5′UTR pairing → translation block	OmrA/B	*E. coli*, *E. amylovora*	[49,50,68]
*flgM*	Anti-sigma28	CDS pairing → translation inhibition	OmrA/B	*E. coli*	[50]
*fliC*	Flagellin	Indirect via *flgM* repression	OmrA/B indirect	*E. coli*	[50]
*csgD*	Curli/biofilm regulator	Upstream RBS pairing, remodels SL1 of *csgD* leader → translation inhibition	OmrA/B	*E. coli*	[54,55]
*dgcM*	Diguanylate cyclase	RBS pairing, OmrA binds extra site → translation inhibition	OmrA/B	*E. coli*	[60]
*dgcT*	Diguanylate cyclase	Codon pairing near AUG, RelA-dependent → translation inhibition	OmrB	*E. coli*	[63,65]
*rydC*	sRNA regulator	OmrB “sponge” action	OmrB	*E. coli*	[87]
*cfa*	FA synthase	Indirect via RydC sequestration, OmrB “sponge” action	OmrB indirect	*E. coli*	[87]
*acrA*	Efflux pump	Synthetic OmrB scaffold → translation inhibition	Synthetic	*E. coli*	[88]
*kvrA*	Capsule regulator	Post-transcriptional repression	OmrB direct; possibly OmrA	*K. pneumoniae*	[71]
*LEE genes*	T3SS effectors	Indirect via LEE1	OmrA/B indirect	EHEC/EPEC	[78]
*ler*	LEE master regulator	Indirect (no base pairing)	OmrA/B	EHEC/EPEC	[78]

## Data Availability

No new data were created or analyzed in this study. Data sharing is not applicable to this article.
